# Matrix metallopeptidase expression and modulation by transforming growth factor-β1 in equine endometrosis

**DOI:** 10.1038/s41598-020-58109-0

**Published:** 2020-01-24

**Authors:** Anna Szóstek-Mioduchowska, Mariola Słowińska, Joanna Pacewicz, Dariusz J. Skarzynski, Kiyoshi Okuda

**Affiliations:** 1 Department of Reproductive Immunology and Pathology, Institute of Animal Reproduction and Food Research, Polish Academy of Sciences, 10-748 Olsztyn, Poland; 2 Department of Gamete and Embryo Biology,Institute of Animal Reproduction and Food Research, Polish Academy of Sciences, 10-748 Olsztyn, Poland; 3 Laboratory of Reproductive Physiology Graduate School of Natural Science and Technology, Okayama University, 700-8530 Okayama, Japan; 4 Obihiro University of Agriculture and Veterinary Medicine, Obihiro, Japan

**Keywords:** Chronic inflammation, Infertility

## Abstract

Equine endometrial fibrosis (endometrosis) is described as a degenerative chronic condition in the uterus. Its characteristic feature is excessive deposition of extracellular matrix (ECM) components around the endometrial glands and stroma. Although matrix metallopeptidases (MMPs) that mediate ECM turnover are important factors in the process of fibrosis, knowledge of their expression and regulation in endometrosis is limited. In other species, one of the important regulators of MMPs and tissue inhibitors of MMPs (TIMPs) is transforming growth factor (TGF)-β1. The goal of this study was to determine (i) endometrial expression of MMPs and TIMPs during endometrosis and (ii) the effect of TGF-β1 on expression of MMPs and TIMPs in equine endometrial fibroblasts and epithelial cells. In the follicular phase of the estrous cycle, MMP-1, -2, -9, and TIMP concentrations were higher during endometrosis than in healthy endometrium (P < 0.05). In the midluteal phase, MMP-3 concentration was lower in severe endometrosis compared to healthy endometrium (P < 0.05). In fibroblasts, TGF-β1 upregulated MMP-1, -9, -13, and TIMP1, but downregulated MMP-3 secretion (P < 0.05). In epithelial cells, TGF-β1 upregulated MMP-1, -9, -13, and TIMP secretion (P < 0.05). Endometrial expression of MMPs and TIMPs is altered during endometrosis. TGF-β1 is a regulator of endometrial ECM remodeling via its effect on MMPs and TIMPs in equine endometrial fibroblasts and epithelial cells.

## Introduction

Equine endometrial fibrosis (endometrosis) is a chronic degenerative condition in the uterus, and is described as an active or inactive fibrosis that develops around the endometrial glands and/or in the stroma^1^. The term endometrosis was introduced by Kenney^2^ to define changes in the mare uterus previously referred to as chronic degenerative endometritis. In contrast to endometrosis, the term endometriosis defines a condition involving extra-uterine implantation of endometrial tissue in women. Endometrial fibrosis in mares is associated with pathological changes in the endometrial glands, such as cystic dilation and atrophy or hypertrophy of the epithelium^1^. Excessive deposition of extracellular matrix (ECM) components such as collagen type I (COL1) and fibronectin (FN) around the endometrial glands and stroma is a characteristic feature of this condition. In mare endometrial fibrosis, tissue architecture is destroyed and endometrial functions are altered, resulting in death and loss of the embryo^3–7^. The severity of mare endometrosis is classified based only on histological assessment of endometrium and the magnitude of periglandular fibrosis and inflammatory changes^1^. Depending on the degree of endometrial structural changes, endometrial fibrosis in mares is divided into four stages as follows: I (healthy endometrium, no fibrosis), IIA (mild fibrosis), IIB (moderate fibrosis), and III (severe fibrosis) according to Kenney and Doig^1^. The reproductive outcome depends on the stage of endometrial fibrosis in mares. The foaling rates in category I, IIA, IIB, and III endometria are 80%–90%, 50%-80%, 10%-50%, and 10%, respectively^1^. Thus, endometrial fibrosis is a serious problem in horse reproduction and poses a great economic loss to the horse-breeding industry. A greater understanding of the pathogenesis of endometrosis may contribute to the creation of new strategies for prevention and treatment of this condition.

In healthy tissue, there is a balance between synthesis and degradation of collagen that is achieved by orchestrated production of cytokines, growth factors, and matrix metallopeptidases (MMPs). In fibrotic tissue, there is excessive deposition of ECM components. Matrix metallopeptidases are a group of zinc-dependent endopeptidases that mediate ECM turnover. The endogenous inhibitors of MMPs are inhibitors of metallopeptidases (TIMPs). Although MMPs are important factors in the process of fibrosis, knowledge of their expression and regulation in endometrial fibrosis in mares is limited^8–10^. Recently, we showed that MMPs and TIMPs are regulated by interleukin (IL)−1β and IL-6 during endometrial fibrosis in mares^10^. Additionally, MMPs have been shown to be modulated by transforming growth factor (TGF)-β1 in human and murine endometrial cells and tissue^11–13^. This cytokine seems to be a key molecule that shows multifaceted roles in the pathogenesis of fibrosis^14–16^. To the best of our knowledge, the role of TGF-β1 in processes related to pathogenesis of equine endometrial fibrosis is not well known. The endometrial concentration of TGF-β1 is correlated with the progression of fibrosis in mares^17^. Recently, we showed that TGF‐β1 effects equine endometrial fibroblast proliferation, collagen synthesis, and myofibroblast differentiation^18^. However, the mechanism underlying regulation of MMP expression by TGF-β1 in equine endometrial cells has not yet been established.

The goal of this study was to develop a better understanding of (i) the expression profile of endometrial MMPs and TIMPs in the course of equine endometrial fibrosis, and (ii) the effect of TGF-β1 on endometrial ECM remodeling through the expression of MMPs and TIMPs in equine endometrial fibroblasts and epithelial cells.

## Results

### Experiment 1. The endometrial MMPs and their tissue inhibitors in the development of endometrial fibrosis in mares

#### MMP-1

**Figure 1:**
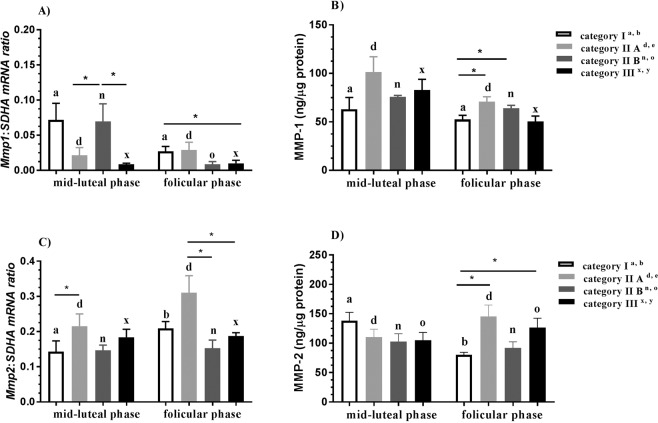
Expression of MMP-1 and -2 in endometrium during mare endometrial fibrosis. Endometrial *Mmp1* mRNA transcription (**A**), MMP-1 concentration (**B**), *Mmp2* mRNA transcription (**C**), and MMP-2 concentration (**D**) in the midluteal phase and follicular phase of the estrous cycle in the progress of mare endometrial fibrosis (Kenney and Doig’s endometrium categories I, IIA, IIB and III) in equine endometrium. Superscript letters indicate statistical differences between the midluteal and follicular phases in Kenney and Doig’s category I^a,b^, IIA^d,e^, IIB^n,o^, and III^x,y^. Asterisks indicate statistical differences between *Mmp1* and *Mmp2* mRNA transcription/protein expression during mare endometrial fibrosis within the midluteal or follicular phases (*P < 0.05; **P < 0.01).

In category IIB endometrium, *Mmp1* mRNA transcription was upregulated in the midluteal phase as compared to the follicular phase of the estrous cycle (P < 0.05; Fig. 1A). Additionally, in the midluteal phase of the estrous cycle, *Mmp1* mRNA transcription was upregulated in category IIB endometrium as compared to category IIA and III endometria (P < 0.05 and P < 0.05, respectively; Fig. 1A). In the follicular phase of the estrous cycle, *Mmp1* mRNA transcription was downregulated in category III endometrium as compared to category I endometrium (P < 0.05, Fig. 1A). In turn, in the follicular phase, MMP1 concentration was higher in category IIA and IIB endometria than in category I endometrium (both P < 0.05; Fig. 1B).

#### MMP-2

In the midluteal phase of the estrous cycle, *Mmp2* mRNA transcription was upregulated in category IIA endometrium as compared to category I endometrium (P < 0.05; Fig. 1C). In the follicular phase, *Mmp2* mRNA transcription was downregulated in IIB and III endometrium as compared to category IA endometrium (both P < 0.05; Fig. 1C). In the follicular phase of the estrous cycle, MMP-2 concentration was higher in category IIA and III endometrium as compared to category I endometrium (both P < 0.05; Fig. 1D). In category I endometrium, MMP-2 concentration was higher in the midluteal phase compared to the follicular phase of the estrous cycle (P < 0.05; Fig. 1D).

#### MMP-3

**Figure 2:**
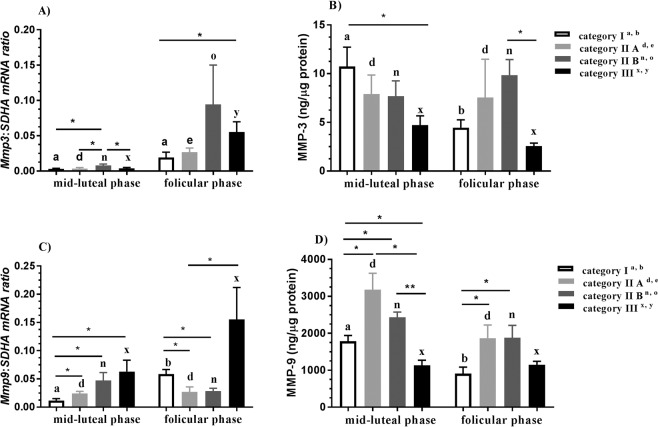
Expression of MMP-3 and -9 in endometrium during mare endometrial fibrosis. Endometrial *Mmp3* mRNA transcription (**A**) and MMP-3 concentration (**B**) and *Mmp9* mRNA transcription (**C**) and MMP-9 concentration (**D**) in the midluteal phase and follicular phase of the estrous cycle in the progression of mare endometrial fibrosis (Kenney and Doig’s endometrium categories I, IIA, IIB and III) in equine endometrium. Asterisks indicate statistical differences between *Mmp3* and *Mmp9* mRNA transcription/protein expression during mare endometrial fibrosis, within the midluteal or follicular phases (*P < 0.05; **P < 0.01).

In category IIA, IIB, and III endometria, *Mmp3* mRNA transcription was downregulated in the midluteal phase as compared to the follicular phase of the estrous cycle (P < 0.01, P < 0.05, and P < 0.01, respectively; Fig. 2A). Additionally, in the midluteal phase of the estrous cycle, *Mmp3* mRNA transcription was upregulated in category IIB endometrium as compared to category I, IIA, and III endometria (P < 0.05; Fig. 2A). In the follicular phase of the estrous cycle, *Mmp3* mRNA transcription was upregulated in category III endometrium as compared to category I (P < 0.05, Fig. 2A). In category I endometrium, MMP-3 concentration was higher in the midluteal phase than in the follicular phase (P < 0.05; Fig. 2B). In turn, in the midluteal and follicular phases, MMP-3 concentration was lower in category III endometrium than in category I and IIB endometria, respectively (P < 0.05, Fig. 2B).

#### MMP-9

In category I endometrium, *Mmp9* mRNA transcription was downregulated in the midluteal phase compared to the follicular phase of the estrous cycle (P < 0.01; Fig. 2C). Additionally, in the midluteal phase, *Mmp9* mRNA transcription was upregulated in category IIA, IIB, and III endometria as compared to category I endometrium (P < 0.05; Fig. 2C). In the follicular phase of the estrous cycle, *Mmp9* mRNA transcription was downregulated in category IIA and IIB endometria as compared to category I endometrium (both P < 0.05; Fig. 2C) and *Mmp9* mRNA transcription was downregulated in category IIA endometrium as compared to category III endometrium (P < 0.05; Fig. 2C). In the midluteal phase of the estrous cycle, MMP-9 concentration was higher in category IIA and IIB endometria as compared to category I endometrium (both P < 0.05; Fig. 2D). Additionally, in the midluteal phase, MMP-9 concentration was lower in category III endometrium as compared to category I, IIA and IIB endometria (P < 0.05, P < 0.05 and P < 0.01, respectively; Fig. 2D). In the follicular phase, MMP-9 concentration was higher in category IIA and IIB endometria as compared to category I endometrium (both P < 0.05; Fig. 2D).

#### MMP-13

**Figure 3:**
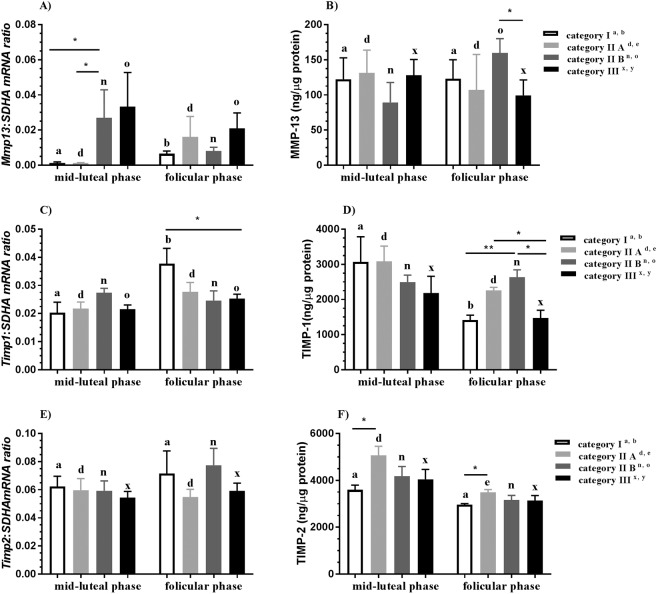
Expression of MMP-13, TIMP-1, and TIMP-2 in endometrium during mare endometrial fibrosis. Endometrial *Mmp13* mRNA transcription (**A**) and MMP-13 concentration (**B**), *Timp1* mRNA transcription (**C**) and TIMP-1 concentration (**D**), and *Timp2* mRNA transcription (**E**), TIMP-2 concentration (**F**) in the midluteal phase and follicular phase of the estrous cycle in the progression of mare endometrial fibrosis (Kenney and Doig’s endometrium categories I, IIA, IIB and III) in equine endometrium. Asterisks indicate statistical differences between *Mmp13*, *Timp1*, and *Timp2* mRNA transcription/protein expression during mare endometrial fibrosis within the midluteal or follicular phases (*P < 0.05; **P < 0.01).

In category I endometrium, *Mmp13* mRNA transcription was downregulated in the midluteal phase as compared to the follicular phase of the estrous cycle (P < 0.05; Fig. 3A). Additionally, in the midluteal phase, *Mmp13* mRNA transcription was upregulated in category IIB endometrium as compared to category I and IIA endometria (both P < 0.05; Fig. 3A). The concentration of MMP-13 was higher in category IIB endometrium in the follicular phase than in the midluteal phase (P < 0.05; Fig. 3B). In the follicular phase of the estrous cycle, MMP-13 concentration was higher in category IIB endometrium as compared to category III endometrium (P < 0.05; Fig. 3B).

#### TIMP-1

In category I endometrium, *Timp1* mRNA transcription was downregulated in the midluteal phase as compared to the follicular phase of the estrous cycle (P < 0.05; Fig. 3C). Additionally, in the follicular phase, *Timp1* mRNA transcription was downregulated in category III endometrium as compared to category I endometrium (P < 0.05; Fig. 3C). In turn, in the follicular phase of the estrous cycle, TIMP-1 concentration was higher in category IIB endometrium than in category I and III endometria (P < 0.01 and P < 0.05, respectively; Fig. 3D) and higher in category IIA endometrium than in category III endometrium (P < 0.05; Fig. 3D).

#### TIMP-2

The concentration of TIMP-2 was lower in category IIA endometrium in the follicular phase as compared to midluteal phase of the estrous cycle (P < 0.05; Fig. 3F). TIMP-2 concentration was higher in category IIA endometrium than in category I endometrium in the midluteal and follicular phase of the estrous cycle (both P < 0.05; Fig. 3F). In category I endometrium, TIMP-2 concentration was higher in the midluteal phase than in the follicular phase of the estrous cycle (P < 0.05; Fig. 3F).

### Experiment 2. The effect of TGF-β1 on MMPs and TIMPs in equine endometrial cells *in vitro*

The basal secretion levels of MMPs and TIMPs from epithelial cells and fibroblast are shown in Table 1.

**Table 1:** Basal secretion of MMPs and TIMPs after 24 h and 48 h (control groups) by endometrial fibroblasts and epithelial cells.

	Fibroblasts	
	24 h	48 h
**MMP-1** (pg/μg protein)	263.5 ± 47.72	203.9 ± 64.65
**MMP-2** (pg/μg protein)	3981 ± 168.9	4512 ± 350.1
**MMP-3** (pg/μg protein)	170.9 ± 30.47	237.4 ± 16.28
**MMP-9** (pg/μg protein)	1462.5 ± 43.1	1662.4 ± 12.68
**MMP-13** (pg/μg protein)	1390.5 ± 84.41	1964.4 ± 134.8
TIMP-1 (pg/μg protein)	222.2 ± 20.59	257.3 ± 16.56
**TIMP-2** (pg/μg protein)	72.54 ± 3.302	84.84 ± 2.648
	**Epithelial cells**	
	**2**4 **h**	**48** **h**
**MMP-1** (pg/μg protein)	416.3 ± 92.14	463.6 ± 93.37
**MMP-2** (pg/μg protein)	7015 ± 1711	11390 ± 1909
**MMP-3** (pg/μg protein)	231.4 ± 87.34	209.3 ± 75.55
**MMP-9** (pg/μg protein)	1375 ± 692.9	795.9 ± 228.4
**MMP-13** (pg/μg protein)	7081 ± 1815	27840 ± 1464
**TIMP-1** (pg/μg protein)	659.8 ± 58.73	780.2 ± 75.47
**TIMP-2** (pg/μg protein)	170 ± 73.61	189.1 ± 42.62

### Effect of TGF-β1 on fibroblasts

**Figure 4:**
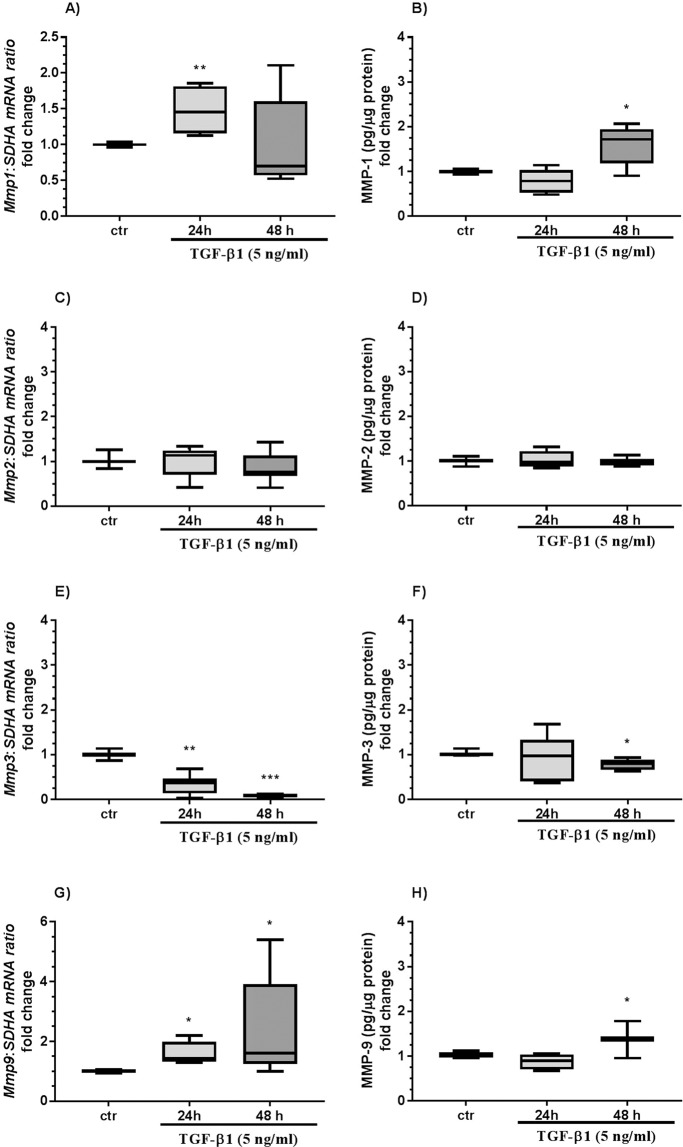
The effect of TGF-β1 on MMP-1, -2, -3, and -9 in equine endometrial fibroblasts. The effect of TGF-β1 (5 ng/ml) on *Mmp1* mRNA transcription (**A**) and MMP-1 secretion (**B**), *Mmp2* mRNA transcription (**C**) and MMP-2 secretion (**D**), *Mmp3* mRNA transcription (**E**) and MMP-3 secretion (**F**), and *Mmp9* mRNA transcription (**G**) and MMP-9 secretion (**H**) from endometrial fibroblasts cultured *in vitro* (n = 6) for 24 h and 48 h. All values are expressed as a fold change. Asterisks indicate statistical differences (*P < 0.05) from the respective control, as determined by a nonparametric Mann-Whitney U test.

**Figure 5:**
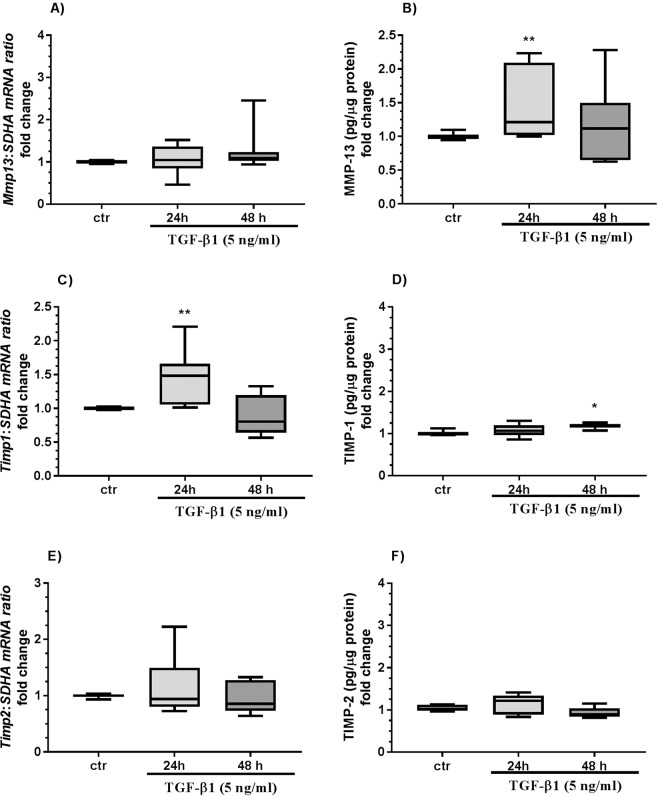
The effect of TGF-β1 on MMP-13 and TIMP-1 and -2 in equine endometrial fibroblasts. The effect of TGF-β1 (5 ng/ml) on *Mmp13* mRNA transcription (**A**) and MMP-13 secretion (**B**), *Timp1* mRNA transcription (**C**) and TIMP-1 secretion (**D**), and *Timp2* mRNA transcription (**E**) and TIMP-2 (**F**) secretion from endometrial fibroblasts cultured *in vitro* (n = 6) for 24 and 48 h. All values are expressed as a fold change. Asterisks indicate statistical differences (*P < 0.05) from the respective control, as determined by a nonparametric Mann-Whitney U test.

**Figure 6:**
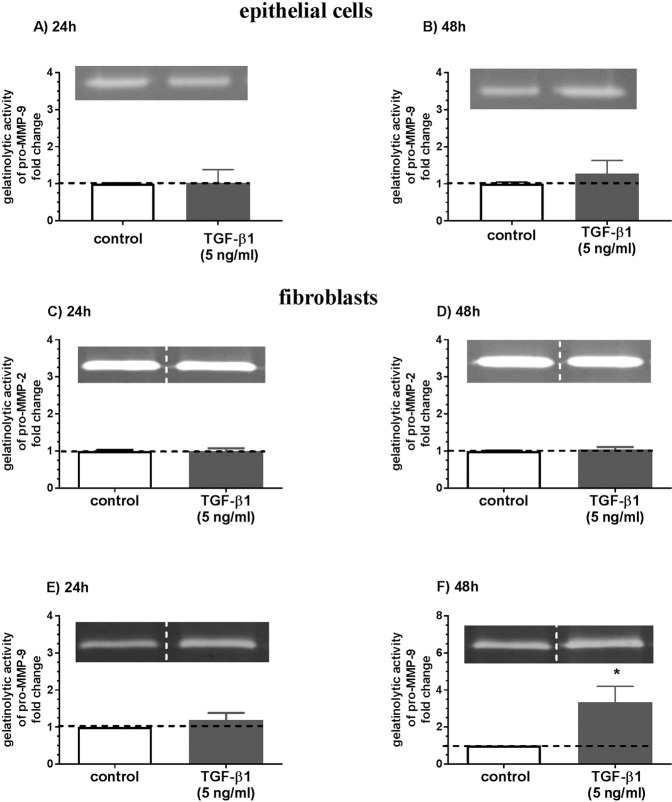
The effect of TGF-β1 on pro-MMP-2 and pro-MMP-9 gelatinolytic activity in equine endometrial fibroblasts and epithelial cells. The effect of TGF-β1 (5 ng/ml) on pro-MMP-2 and pro-MMP-9 gelatinolytic activity in endometrial epithelial cells (**A,B**) (n = 5) and fibroblasts (**C–F**; n = 6) cultured *in vitro*. The dotted line indicates the place where blots from the same gel were grouped. All values are expressed as a fold change. Asterisks indicate statistical differences (*P < 0.05) from the respective control, as determined by a nonparametric Mann-Whitney U test.

Transforming growth factor-β1 treatment upregulated *Mmp1* and *Timp1* mRNA transcription after 24 h (Figs. 4A and 5C; both P < 0.01) and MMP-1 and TIMP-1 secretion after 48 h from endometrial fibroblasts (Figs. 4B and 5D; both P < 0.05) as compared to the respective control groups. TGF-β1 treatment decreased *Mmp3* mRNA transcription after 24 h and 48 h (Fig. 4E; P < 0.01 and P < 0.001, respectively) and MMP-3 secretion after 48 h (Fig. 4F; P < 0.05). TGF-β1 treatment increased *Mmp9* mRNA transcription after 24 h and 48 h (Fig. 4G; P < 0.05) and MMP-9 secretion (Fig. 4H; P < 0.05) and pro-MMP-9 gelatinolytic activity (Fig. 6F; P < 0.05) after 48 h.

Transforming growth factor-β1 treatment increased MMP-13 secretion after 24 h (Fig. 5B; P < 0.01).

### Effect of TGF-β1 on epithelial cells

**Figure 7:**
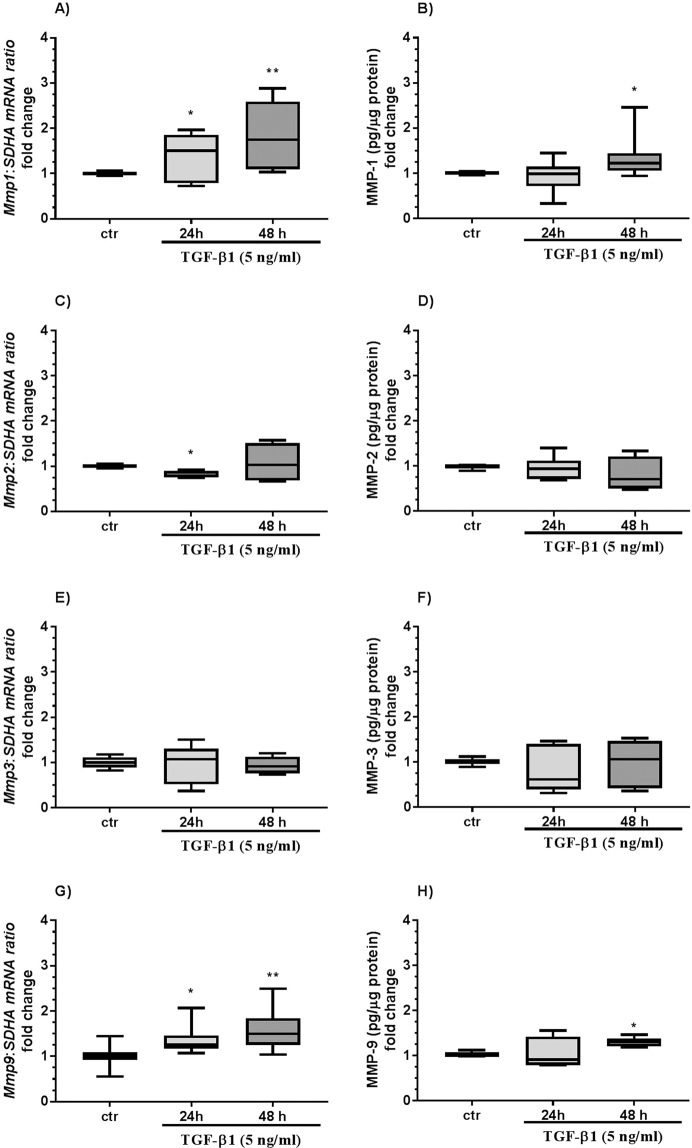
The effect of TGF-β1 on MMP-1, -2, -3, and -9 in equine endometrial epithelial cells. The effect of TGF-β1 (5 ng/ml) on *Mmp1* mRNA transcription (**A**) and MMP-1 secretion (**B**), *Mmp2* mRNA transcription (**C**) and MMP-2 secretion (**D**), *Mmp3* mRNA transcription (**E**) and MMP-3 secretion (**F**), and *Mmp9* mRNA transcription (**G**) and MMP-9 secretion (**H**) from endometrial epithelial cells cultured *in vitro* (n = 5) for 24 and 48 h. All values are expressed as a fold change. Asterisks indicate statistical differences (*P < 0.05) from the respective control, as determined by a nonparametric Mann-Whitney U test.

**Figure 8:**
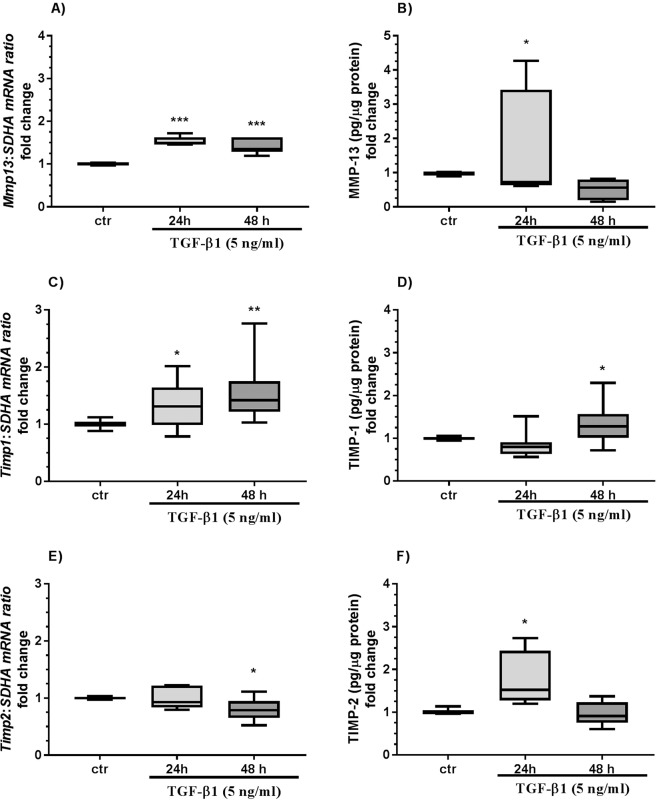
The effect of TGF-β1 on MMP-1, -2, -3, and -9 in equine endometrial epithelial cells. The effect of TGF-β1 (5 ng/ml) on *Mmp13* mRNA transcription (**A**) and MMP-13 secretion (**B**), *Timp1* mRNA transcription (**C**) and TIMP-1 secretion (**D**), and *Timp2* mRNA transcription (**E**) and TIMP-2 secretion (**F**) from endometrial epithelial cells cultured *in vitro* (n = 5) for 24 and 48 h. All values are expressed as a fold change. Asterisks indicate statistical differences (*P < 0.05) from the respective control, as determined by a nonparametric Mann-Whitney U test.

Transforming growth factor-β1 treatment increased *Mmp1, Mmp9*, and *Mmp13* mRNA transcription after 24 h and 48 h (Figs. 7A,G; 8A; P < 0.05) and MMP-1 and MMP-9 secretion after 48 h (Figs. 7B,H; P < 0.05) and MMP-13 secretion from epithelial cells after 24 h (Fig. 8B; P < 0.05) as compared to the control group. Additionally, TGF-β1 treatment decreased *Mmp2* mRNA transcription after 24 h (Fig. 7C; P < 0.05), and increased *Timp1* mRNA transcription after 24 h and 48 h (Fig. 8C; P < 0.05; P < 0.01) and TIMP-1 secretion after 48 h (Fig. 8D; P < 0.05). In turn, TGF-β1 treatment decreased *Timp2* mRNA transcription after 48 h (Fig. 8E; P < 0.05), but increased TIMP-2 secretion after 24 h (Fig. 8F; P < 0.05). Pro-MMP-2 gelatinolytic activity after TGF-β1 treatment was not detectable in epithelial cells.

## Discussion

Matrix metallopeptidases play an important role in many physiological processes, such as angiogenesis, inflammation, ovulation, and embryogenesis. Additionally, MMPs are involved in cyclic changes in structure and thickness of the endometrium. However, dysregulated expression of various MMPs is associated with many pathological processes, such as fibrosis, weakening of ECM or tissue destruction, e.g., in cancer metastasis^19,20^. Thus, a balance between activation and inhibition of MMPs is crucial for maintaining tissue homeostasis. To the best of our knowledge, this study shows for the first time estrous–phase-dependent differences in the expression of MMPs and their tissue inhibitors in the course of endometrial fibrosis in mares.

Data concerning MMP expression in equine endometrium in the course of endometrial fibrosis are limited. Walter *et al*.^8^ showed by western analysis and zymography that the active form of MMP-2 was upregulated in mare fibrotic endometrium. In turn, Aresu *et al*.^9^ used immunohistochemistry to demonstrate that there were no differences in expression of MMP-2 and MMP-9 between healthy endometrium and endometrium effected by fibrosis in mares. Additionally, Centeno *et al*.^21^ showed that transcription of *Mmp1* mRNA was downregulated and transcription of *Mmp2* was upregulated during severe mare endometrial fibrosis. Our studies are in agreement with those of Walter *et al*.^8^ and partially with those of Centeno *et al*.^21^. We showed that endometrial transcription of *Mmp1*, 2, *3, 9*, 13 and *Timp1* and 2 mRNA differ in the course of endometrial fibrosis in mares. These findings suggest that significant differences in endometrial MMP and TIMP expression during endometrial fibrosis reflect complex alterations that take place in ECM. The elevated level of MMPs during endometrial fibrosis in mares is undisputed, but the precise sequence of events in the pathogenesis of this condition is unclear. It remains ambiguous if changes in their activity and expression appear primarily in the endometrium or if specific conditions in the endometrium, such as inflammation or other immunological processes, alter MMP activity. The elevated level of MMPs may also be a cellular response to excessive ECM production and disruption in the normal regulation of endometrial MMP expression. For a long time, MMPs were considered to be principally responsible for turnover and degradation of ECM substrates. However, the action of MMPs is not limited to effects on ECM turnover but also extend to cellular activities, such as cell proliferation and survival, gene expression, and multiple aspects of inflammation that impact outcomes related to fibrosis^22^. Thus, elevated levels of endometrial MMP in the course of mare endometrial fibrosis suggest that MMPs, in addition to their proteolytic function, can contribute to modification of the endometrial microenvironment, enhancing fibrosis. The elevated level of MMPs may be associated with TGF-β1 and other MMP activation^23–27^, myofibroblast differentiation^28^, and cell proliferation and contractility^25,29^.

Changes in the expression profile of MMPs and TIMPs have been shown to be associated with fibrosis in many organs and their expression profiles seems to be highly tissue-specific [reviewed in^30,31^]. Additionally, the dysregulated profile of MMP and TIMP expression was observed in human endometriosis^32–34^, and inhibition of MMPs suppresses the development of this disease^12,35^. Despite obvious differences, mare endometrosis and endometriosis in women have more in common than has been considered before. Vigano *et al*.^36^ proposed redefinition of the term endometriosis and suggested a change in definition from “presence of endometrial epithelial and stromal cells at ectopic sites” to “a fibrotic condition in which endometrial stroma and epithelium can be identified at ectopic sites”. Another uterine disorder with signs of fibrosis is adenomyosis, which is characterized by abnormal presence of endometrial tissue in the myometrium. The main mechanisms involved in adenomyosis pathogenesis include aberrations in sex steroid hormone functions, dysregulated cell proliferation and fibrosis, inflammation, and neuroangiogenesis^37^. Currently, the best animal models to investigate human endometriosis and adenomyosis include autologous or syngeneic rodent models, xenotransplantation of human endometrium into immunodeficient mouse models, and nonhuman primate animal models of endometriosis^38^. The use of these models is challenging; however, in the context of the proposal of Vigano *et al*.^36^, mare endometrial fibrosis may be a suitable model for understanding pathogenic mechanisms occurring in fibrotic processes in human endometriosis.

Considered the increased expression of MMPs and TGF-β1 in the course of mare endometrial fibrosis^17^ and the fact that TGF-β1 increased the markers of fibrosis, such as COL1, COL3, FN, and α-smooth muscle actin (α-SMA)^18^, we aimed to determine the role of TGFβ-1 in regulation of MMPs and their inhibitors, which are another important markers of fibrosis. Our results suggest that TGF-β1 may augment its own profibrotic action by increasing expression of MMPs. The increase in MMP-1, -9, and -13 in response to TGF-β1 treatment in equine endometrial fibroblasts and epithelial cells, independent of their proteolytic function, seems to be associated with TGF-β1 activation^23–26^, activation of other MMPs^27^, myofibroblast differentiation^28^, and cell proliferation^25,29^, thus enhancing fibrosis. Only activated TGF-1β exerts its biological effect. Matrix metallopeptidases are known to proteolytically activate latent TGF-β1 that is sequestered in the ECM, and activated TGF-β1 in turn may augment its profibrotic action. This shows that there is tight regulatory loop between TGF-β1 and MMPs^23–29^. The complex positive feedback between TGF-β1 and MMPs may play an important role in progression of mare endometrosis. However, further study is needed to elucidate the exact role of MMP in mare endometrial fibrosis. A next step should be to investigate the effect of MMPs and their inhibitors on endometrial cell properties such as proliferation and migration, and activation of other factors such as TGF-β1, IL-1β, pro-MMP-2, pro-MMP-9, TNF-α, and myofibroblast differentiation.

The molecular mechanism underlying TGF-β1 action on MMP and TIMP expression in mare endometrial cells has not yet been identified and has to be clarified in further studies. Transforming growth factor-β1 is a pleiotropic cytokine and activates many intracellular signaling pathways, which is a reason for its wide role in physiological and pathological processes and its effect on MMP and TIMP expression. However, it is known that TGF-β1 may induce fibrosis by activating MMPs by noncanonical pathways through p38 MAP kinase (p38 MAPK), PI3K, and JNKs^39^. Similarly, in highly invasive breast cancer cells, TGF-β1 affects MMP and TIMP expression through p38 MAPK and ERK1/2 pathways^40^. TGF-β1 stimulates MMP-2 expression through the activation of the Rac1/ROS/NFκB pathway and thus increases invasiveness of SW1990 human pancreatic cancer cells^41^. Additionally, in transformed keratinocytes and breast cells, TGF-β1 increases MMP-9 expression by the activation of Rac1/ROS/NFκB and TAK1-NFκB pathways^42,43^.

Elevated levels of MMPs and TIMPs in mare endometrial fibrosis indicate that MMPs are potential therapeutic targets for mare endometrial fibrosis in veterinary medicine. Based upon clinical studies showing increasing concentration of MMP-1, -7, -8, and -9 in idiopathic pulmonary fibrosis (IPF) in blood and lung samples, targeting MMPs and their inhibitors may be new therapeutic approaches for IPF (reviewed in^44^). As reviewed previously, approaches associated with MMPs having potential as therapeutic targets for fibrosis include: small-molecule hydroxymate inhibitors that chelate the Zn^2+^ ion at the active site, monoclonal antibodies blocking MMP activity, antisense nucleic acids that bind and silence mRNA molecules or ribosomes, and activity-based probes that bind and only inhibit active MMPs. Also included are novel biomaterials, such as injectable hydrogels that release specific inhibitors upon proteolytic degradation by the specific active MMP being targeted and interfering with upstream inducers of MMP activity. Another strategy would be to augment the expression of antifibrotic MMPs in fibrotic tissue^44^. However, further functional studies concerning MMPs as a therapeutic target in treatment of mare endometrial fibrosis are needed.

## Conclusion

Endometrial expression of MMPs and TIMPs is altered during mare endometrial fibrosis. Knowledge of factors responsible for activating and inhibiting MMP expression allows a better understanding of ECM remodeling and pathogenesis of fibrosis and will contribute to the development of new drugs targeting MMPs. TGF-β1 is a regulator of endometrial ECM remodeling via its effect on MMPs and their tissue inhibitors in fibroblasts and epithelial cells. TGF-β1 appears to enhance its own profibrotic action by affecting MMPs and their inhibitors. The upregulation of MMP-1, -9, and -13 by TGF-β1 in equine endometrial fibroblasts and epithelial cells, independent of their proteolytic function, appears to be connected to TGF activation, myofibroblast differentiation, and cell proliferation, thereby augmenting fibrosis. In turn, lower levels of MMP-3 may be associated with inhibition of ECM degradation during the process of fibrosis.

## Materials and Methods

### Tissue collection

Uteri (n = 51) were obtained post-mortem from mares with an estrous cycle at a local slaughterhouse (Rawicz, Poland (Experiment 1); Kumamoto, Japan (Experiment 2). To be sure that an adequate number of mares would be available within each experimental group, about 160 endometrial samples were collected over the whole mare reproductive season (April to July). To carry out hematoxylin-and-eosin staining, pieces of endometrial tissue were placed in 4% buffered paraformaldehyde^45^. Then, endometria were classified microscopically as category I, IIA, IIB, or III according to the Kenney and Doig classification^1^. Together with assessment of the estrous cycle phase, n samples were selected randomly from each category for the first experiments. The materials collected were reviewed and accepted following the guidelines of the Local Ethics Committee for Experiments on Animals in Olsztyn, Poland (Agreements No. 51/2011; Experiment 1) and the Local Institutional Animal Care and Use Committee in Japan (Experiment 2). Declaration of official government veterinary inspection as well as individual veterinary history of the health of the animals confirmed that mares were clinically healthy. The animals were slaughtered in order to obtain meat as part of routine breeding as slaughter animals. Samples of peripheral blood were collected into heparinized tubes immediately before slaughter for progesterone (P_4_) analysis. Based on P_4_ analysis and macroscopic observation of the ovaries, the phases of the estrous cycle were identified^45^. Corpora hemorrhagica presence and blood plasma concentration of P_4_ >1 ng/ml indicated early luteal phase. The presence of a well-developed corpus luteum (CL), follicles 15–20 mm in diameter, and blood plasma concentration of P_4_ >6 ng/ml indicated the midluteal phase. The absence of an active CL and the presence of a follicle >35 mm in diameter, with blood plasma concentration of P_4_ <1 ng/ml indicated the follicular phase. Uteri were obtained within 5 min. of animal death.

Healthy endometria without fibrosis were graded as category I; categories IIA, IIB, and III corresponded to mild, moderate, and severe fibrosis, respectively^1^. For endometrial cell isolation, uterine horns were put into sterile, Ca^2+^- and Mg^2+^-free Hanks’ balanced salt solution (HBBS) with gentamicin (20 μg/ml; Sigma-Aldrich, St. Louis, MO) and bovine serum albumin (0.1%; BSA; Sigma-Aldrich; #A9056), kept on ice, and transported quickly to the laboratory.

### Endometrial cell isolation and culture

Fibroblasts and epithelial cells were isolated, cultured, and passed as described previously for epithelial cells^46^ and fibroblasts^18^. Homogeneity of fibroblast and epithelial cell preparations was confirmed using immunofluorescence staining for vimentin and cytokeratin, respectively^46^. The purity of epithelial cell and fibroblast preparations after isolation was approximately 92% and after passaging was 98% for both types of cells.

### Experiment 1. The endometrial MMPs and their tissue inhibitors in the development of endometrial fibrosis in mare

Endometrial tissue samples (n = 40) from the midluteal and follicular phase of the estrous cycle (n = 5 for each category [I, IIA, IIB, III] within each phase of the estrous cycle) were used. The endometrial *Mmp1*, 2, 3*, 9, 13, Timp1*, and *Timp2* mRNA transcription and concentration of MMP-1,-2,-3,-9,-13 and TIMP-1, and TIMP-2 was determined using real-time PCR and ELISA, respectively. Preparation of tissue homogenates for ELISA was carried out according to the manufacturer’s protocols. To normalize the data, the concentration of all proteins was assessed per 1 μg of protein. The protein concentration was quantified using Bicinchoninic Acid Kit (BCA; BCA-1; Sigma-Aldrich).

### Experiment 2. The effect of TGF-β on MMPs and TIMPs in equine endometrial cells *in vitro*

Endometrial tissue samples (n = 11; category I of Kenney and Doig^1^ at the early luteal phase of the estrous cycle) were used. When fibroblast (n = 6) and epithelial cell (n = 5) cultures from passage 1 reached confluence, the culture medium was replaced with fresh Dulbecco’s Modified Eagle Medium (DMEM)/Ham’s F-12 supplemented with gentamicin (20 μg/ml) and BSA (0.1%; w/v) at 38.0 °C in an atmosphere of 5% CO_2_ in air. Fibroblasts and epithelial cells were always treated with vehicle or TGF-β1 (5 ng/ml; T7039; Sigma-Aldrich) for 24 h and 48 h. The dose of TGF-β1 was chosen in a preliminary study. After treatment with TGF-β1, conditioned medium was collected for MMP and TIMP determination using ELISA and MMP-2/9 gelatinolytic activity using zymography. The cells were harvested into TRIZOL Reagent (no. 15596-026; Invitrogen, Carlsbad, CA) and kept at −80 °C until RNA extraction, reverse transcription, and real-time PCR were performed. To normalize MMP and TIMP concentrations, the protein concentration was quantified.

### Analytic methods

#### Real-time PCR

After tissue collection or TGF-β1 treatment, total RNA was extracted from samples using TRIZOL according to the manufacturer’s protocols. The RNA samples were tested using a NanoDrop ND-2000 spectrophotometer and by agarose gel electrophoresis. The optical density A260/280 of RNA samples was approximately 2. The RNA samples were kept at −80 °C. The RNA samples (1 μg) were reverse transcribed into cDNA (QuantiTect Rev. Transcription Kit; no. 205313; Qiagen, Hilden, Germany) following the manufacturer’s protocols. The cDNA samples were kept at −20 °C until real-time PCR was performed.

Real-time PCR analysis was carried out using SYBR Green PCR master mix (Applied Biosystems, Foster City, CA) in the ABI Prism 7900 sequence detection system described recently^18^. The primer sequences for *Mmp1*, *2*, *3*, *9, 13*, *Timp1*, and *Timp2* were previously described^10^. Succinate dehydrogenase complex, subunit A (SDHA) was used as a reference gene. All primers were synthesized by Sigma-Aldrich. The data were analyzed using the method described by Zhao and Fernald^47^.

### Zymography

Gelatinolytic activity of pro-MMP-2 and pro-MMP-9 was detected using zymography, as described^48^. The samples (fibroblasts: 5 μg per well for pro-MMP-2 [n = 6] and pro-MMP-9 gelatinolytic activity [n = 4]; 25 μg per well for pro-MMP-9 [n = 2] [see Supplementary Data 1]; epithelial cells: 40 μg per well) were loaded with nonreducing loading buffer into SDS polyacrylamide (10% v:v) gel polymerized with 0.1% gelatin. After electrophoresis, electrophoretograms were washed twice with 2.5% Triton X-100 for 40 min, incubated in development solution (50 mM Tris-HCl buffer, pH 7.5, containing 200 mM NaCl, 0.02% Triton X-100 with or without 5 mM CaCl_2_) for 24 h at 37 °C. Electrophoretograms were then stained with Coomassie brilliant blue (0.025%) for 24 h and stored in 2% acetic acid. MMP-2 and MMP-9 degrade gelatin present in the acrylamide electrophoretograms. Thus, a clear lysis band indicates the presence of gelatinases and absence of gelatin and. In these samples, two major bands indicating potential gelatinolytic activity were obtained at approximately 92 kDa for the pro-MMP-9 form and at approximately 72 kDa for the pro-MMP-2 form. Molecular weight estimations were made using recombinant Mouse/Rat MMP-2 (R&D Systems, Minneapolis, MN; 924-MP) and Recombinant Human MMP-9 Western Blotting Standard (R&D Systems; WBC018). The gelatinolytic activity was inhibited when electrophoretograms were incubated with development solution containing 5 mM EDTA, which inhibits MMP activity by chelating Ca^2+^. Stimulation by Ca^2+^ and inhibition by EDTA strongly indicated the presence of MMP^49^. The electrophoretograms were photographed using Image Lab Software version 4.0 (Bio-Rad Laboratories, Hercules, CA), and digitized images were stored for further densitometric analysis using ImageJ software (National Institutes of Health, Bethesda, MD).

### Radioimmunoassay

Progesterone was determined by radioimmunoassay (RIA; Diasource, Louvain-la-Neuve, Belgium; KIP1458) with a standard curve ranging from 0.12 to 36 ng/ml.

### ELISA assay

ELISA kits used in the study are listed in Supplementary Table 2. The ranges of standard curves were based on preliminary data and the standard curves were prepared by Cloud-clone. According to experiments the curve ranges for ELISA kits were prepared as customized service. The average intra- and inter-assay coefficients of variation (CVs) for each ELISA kit were 10% and 12%, respectively.

### Statistical analysis

GraphPad Prism 7 software (GraphPad, San Diego, CA) was used for statistical analysis. For each analysis a Gaussian distribution was tested. Parametric analysis was performed, if normal distribution was confirmed. P < 0.05 was considered statistically significant. The data are shown as mean ± standard deviation (S.D). In Experiment 1, two-way ANOVA followed by Bonferroni multiple comparison was performed. In Experiment 2, a nonparametric Mann-Whitney U test was performed. Experiment 2 was performed for epithelial cells 5 times in triplicate and for fibroblasts 6 times in triplicate.

### Ethics approval and consent to participate

The materials collected were reviewed and accepted following the guidelines of the Local Ethics Committee for Experiments on Animals in Olsztyn, Poland (Agreements No. 51/2011; Experiment 1) and the Local Institutional Animal Care and Use Committee in Japan (Experiment 2).

## Supplementary information

Supplementary Table 2.

Supplementary Dataset 1.

## Data Availability

The datasets used and/or analyzed during the current study are available from the corresponding author on reasonable request.
