# Supplementary material for: Matrix metallopeptidase expression and modulation by transforming growth factor-β1 in equine endometrosis
Source: Sci Rep. 2020 Jan 24;10:1119. doi: 10.1038/s41598-020-58109-0 (PMC6981191; doi:10.1038/s41598-020-58109-0)
Supplement: Supplementary file 1 — Supplementary Table 2. [file 41598_2020_58109_MOESM1_ESM.docx]

**Matrix metallopeptidase expression and modulation by transforming growth factor-β1 in equine endometrosis**

**Szóstek-Mioduchowska AZ^1*^, Słowińska M^1^, Pacewicz J^1^, Skarzynski DJ^1^, Okuda K^2,3^**

^1^Department of Reproductive Immunology and Pathology, Institute of Animal Reproduction and Food Research, Polish Academy of Sciences,10-748 Olsztyn, Poland;

^2^Laboratory of Reproductive Physiology Graduate School of Natural Science and Technology, Okayama University, 700-8530 Okayama, Japan;

### ^3^ Obihiro University of Agriculture and Veterinary Medicine, Obihiro, Japan

***** Corresponding author: Dr. Anna Z. Szóstek-Mioduchowska

Institute of Animal Reproduction and Food Research, Polish Academy of Sciences of Olsztyn, Tuwima-st 10, 10-748 Olsztyn, Poland

Tel. (+48) 89 539 31 30

### E-mail: a.szostek-mioduchowska@pan.olsztyn.pl

**Supplementary Table 2. The list of ELISA kits used in the study.**

|  | **ELISA kit Name** | **Organism Species** | **Manufacture, product no. and curve range**  **Experiment 1** | **Manufacture, product no. and curve range**  **Experiment 2** | |
| --- | --- | --- | --- | --- | --- |
| **MMP-1** | ELISA Kit For Matrix Metalloproteinase 1 (MMP-1) | Equus caballus | Cloud-clone; SEA097Eq  0.312-20 ng/ml | | Cloud-clone; SEA097Eq  0.156-5 ng/ml |
| **MMP-2** | ELISA Kit For Matrix Metalloproteinase 2 (MMP-2) | Equus caballus | Cloud-clone; SEA100Eq  1.56- 100 ng/ml | Cloud-clone; SEA100Eq  31.25- 2000 pg/ml | |
| **MMP-3** | ELISA Kit for Matrix Metalloproteinase 3 (MMP-3) | Equus caballus | Cloud-clone; SEA101Eq  31.25-2000 pg/ml | | |
| **MMP-9** | ELISA Kit For Matrix Metalloproteinase 9 (MMP-9) | Equus caballus | Cloud-clone; SEA553Eq  0.625-40 ng/ ml | Cloud-clone; SEA553Eq  15.625-1000 pg/ ml | |
| **MMP-13** | ELISA Kit For Matrix Metalloproteinase 13 (MMP-13) | Equus caballus | Cloud-clone; SEA099Eq  0.312-20 ng/ml | | |
| **TIMP-1** | ELISA Kit for Tissue inhibitors of metalloproteinase 1 (TIMP-1) | Equus caballus | Cloud-clone; SEA552Eq  0.625-40 ng/ ml | Cloud-clone; SEA552Eq  31.25-2000 pg/ ml | |
| **TIMP-2** | ELISA Kit For Tissue inhibitors of metalloproteinase 2 (TIMP-2) | Equus caballus | Cloud-clone; SEA128Eq  7.8-500 ng/ml | Cloud-clone; SEA128Eq  15.6-1000 pg/ ml | |
