# Supplementary material for: Matrix metallopeptidase expression and modulation by transforming growth factor-β1 in equine endometrosis
Source: Sci Rep. 2020 Jan 24;10:1119. doi: 10.1038/s41598-020-58109-0 (PMC6981191; doi:10.1038/s41598-020-58109-0)
Supplement: Supplementary file 2 — Supplementary Dataset 1. [file 41598_2020_58109_MOESM2_ESM.docx]

**Matrix metallopeptidase expression and modulation by transforming growth factor-β1 in equine endometrosis**

**Szóstek-Mioduchowska AZ^1*^, Słowińska M^1^, Pacewicz J^1^, Skarzynski DJ^1^, Okuda K^2,3^**

^1^Department of Reproductive Immunology and Pathology, Institute of Animal Reproduction and Food Research, Polish Academy of Sciences,10-748 Olsztyn, Poland;

^2^Laboratory of Reproductive Physiology Graduate School of Natural Science and Technology, Okayama University, 700-8530 Okayama, Japan;

### ^3^ Obihiro University of Agriculture and Veterinary Medicine, Obihiro, Japan

***** Corresponding author: Dr. Anna Z. Szóstek-Mioduchowska

Institute of Animal Reproduction and Food Research, Polish Academy of Sciences of Olsztyn, Tuwima-st 10, 10-748 Olsztyn, Poland

Tel. (+48) 89 539 31 30

### E-mail: a.szostek-mioduchowska@pan.olsztyn.pl

**Description of Supplementary data 1:**

According to experimental procedure, the bands from electrophoretograms for fibroblast cell culture no. 1 for pro-MMP-9 gelatinolytic activity (control, TGF-β1 – 5ng/ml) for 24h and for 48 h were grouped and shown in Figure 6 in manuscript (Fig. S1; well 2 + well 4; well 6+8) and the band from electrophoretograms for fibroblast cell culture no. 5 for pro-MMP-2 gelatinolytic activity (Fig. S5; control, TGF-β1 – 5ng/ml) for 24h and for 48 h were grouped and shown in Figure 6 in manuscript (well 2 + well 4; well 6+8)

**The scheme for** electrophoretograms for each cell culture (Fig. S1-S6)**. Grouped bands are underline.**

- **Marker for pro-MMP-9 (well 1),**
- **Control 24h (well 2),**
- **TGF-β1 (1 ng/ml) 24 h (well 3),**
- **TGF-β1 (5 ng/ml) 24 h (well 4),**
- **TGF-β1 (10 ng/ml) 24 h (well 5),**
- **Control 48h (well 6),**
- **TGF-β1 (1 ng/ml) 48 h (well 7),**
- **TGF-β1 (5 ng/ml) 48 h (well 8),**
- **TGF-β1 (10 ng/ml) 48 h (well 9),**
- **Control 72h (well 1) (well 10),**
- **TGF-β1 (1 ng/ml) 72 h(well 11),**
- **TGF-β1 (5 ng/ml) 72 h(well 12),**
- **TGF-β1 (10 ng/ml) 72 h (well 13),**
- **Marker for pro-MMP-2 (well 14),**

**
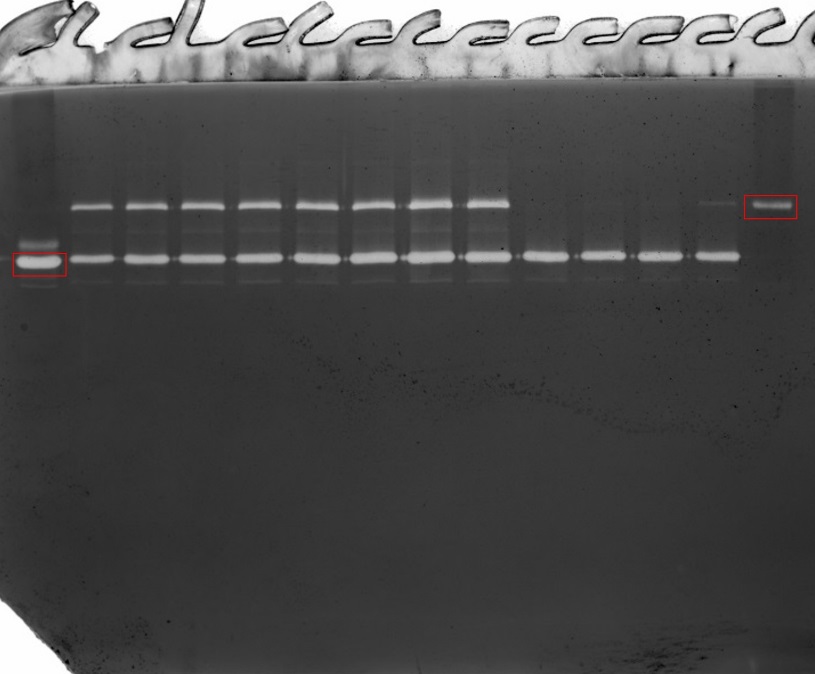
**

**Fig. S1.** Electrophoretograms for fibroblast cell culture no. 1 as above-mentioned scheme, band for markers for pro-MMP-2 and pro-MMP-9 are in red square, respectively. Loaded: 5 ug protein per well.

**
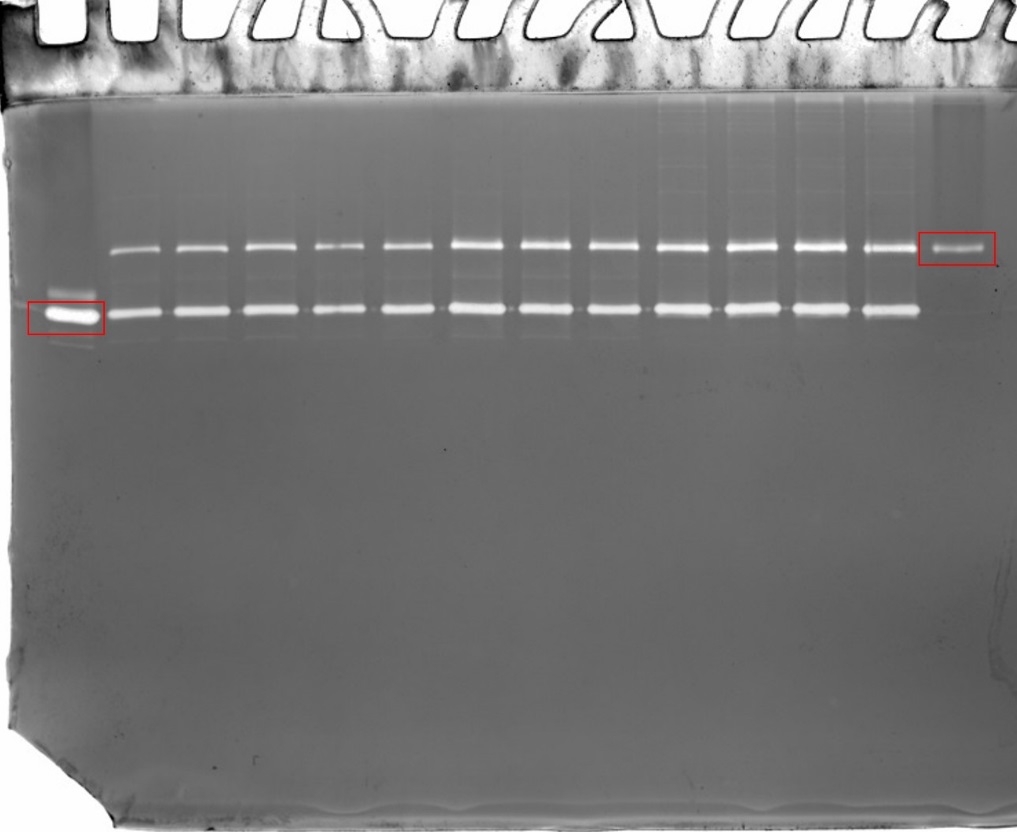
**

**Fig. S2.** Electrophoretograms for fibroblast cell culture no. 2 as above-mentioned scheme, band for markers for pro-MMP-2 and pro-MMP-9 are in red square, respectively. Loaded: 5 ug protein per well.


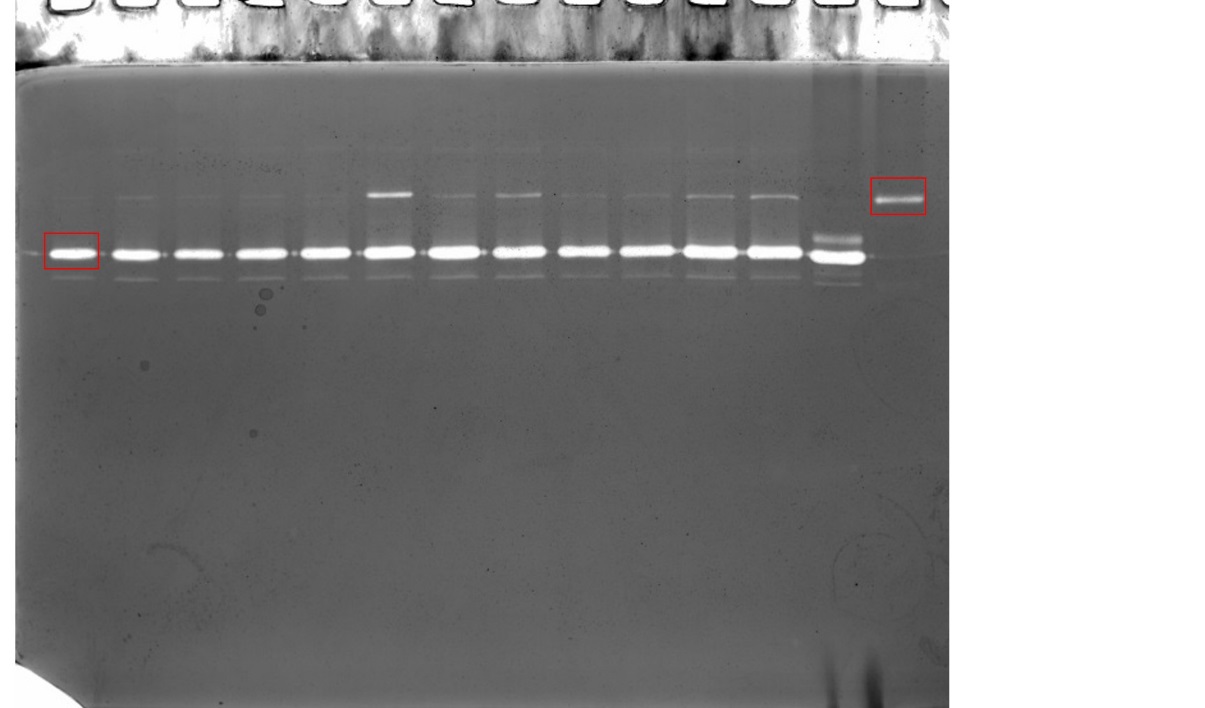


**Fig. S3.** Electrophoretograms for fibroblast cell culture no. 3 as above-mentioned scheme, band for markers for pro-MMP-2 and pro-MMP-9 are in red square, respectively. Loaded: 5 ug protein per well.


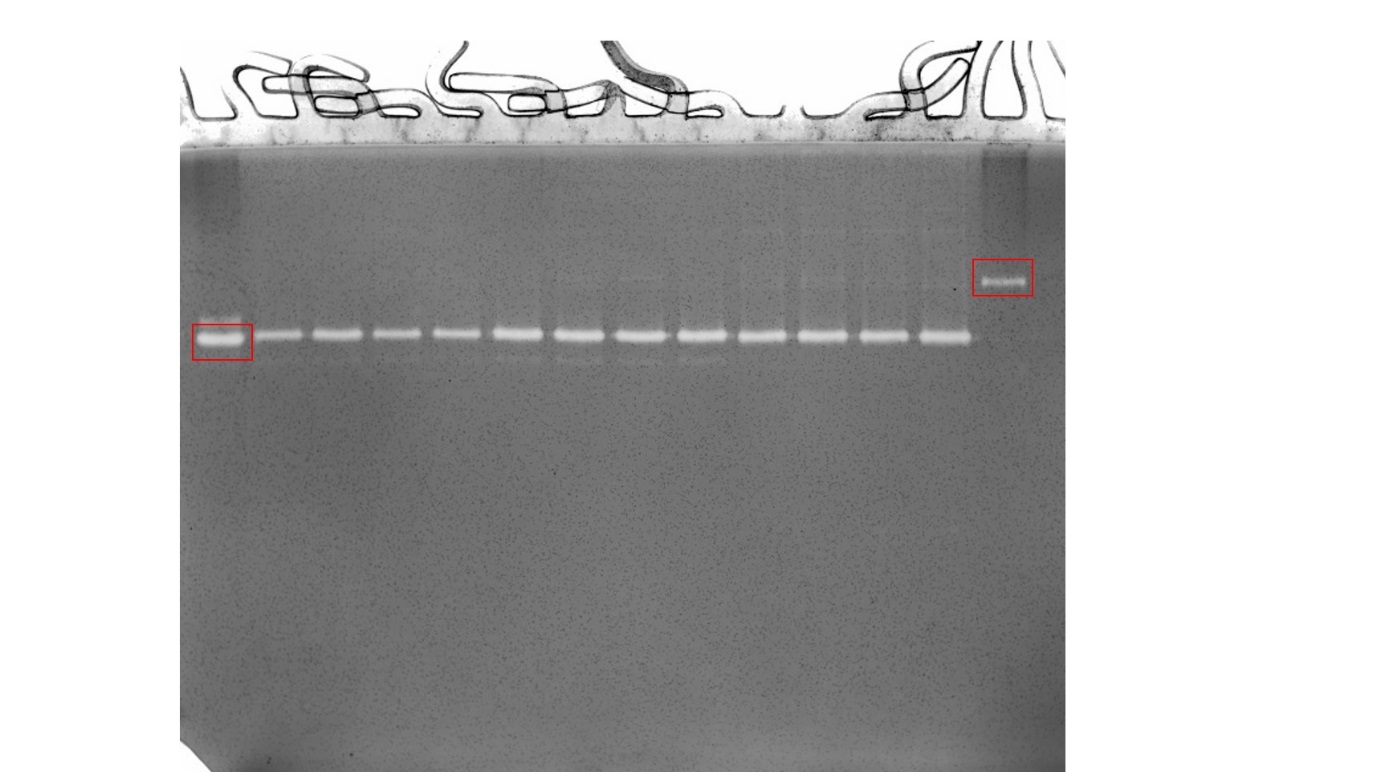


**Fig. S4.** Electrophoretograms for fibroblast cell culture no. 4 as above-mentioned scheme, band for markers for pro-MMP-2 and pro-MMP-9 are in red square, respectively. Loaded: 5 ug protein per well. Zymography was repeated because of low visibility of bands for pro-MMP-9. 25 ug protein per well was loaded. Results below.


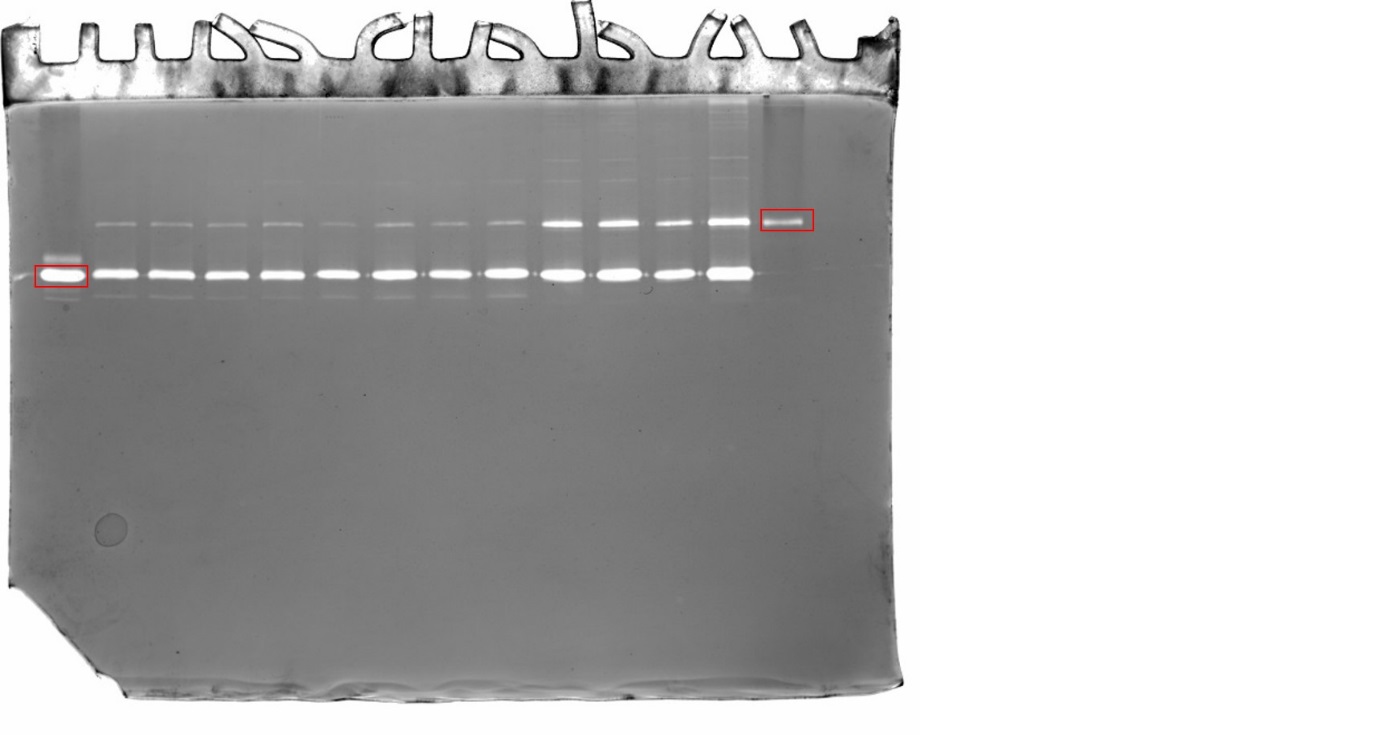


**Fig. S5**. Electrophoretograms for fibroblast cell culture no. 5 as above-mentioned scheme, band for markers for pro-MMP-2 and pro-MMP-9 are in red square, respectively. Loaded: 5 ug protein per well.

**
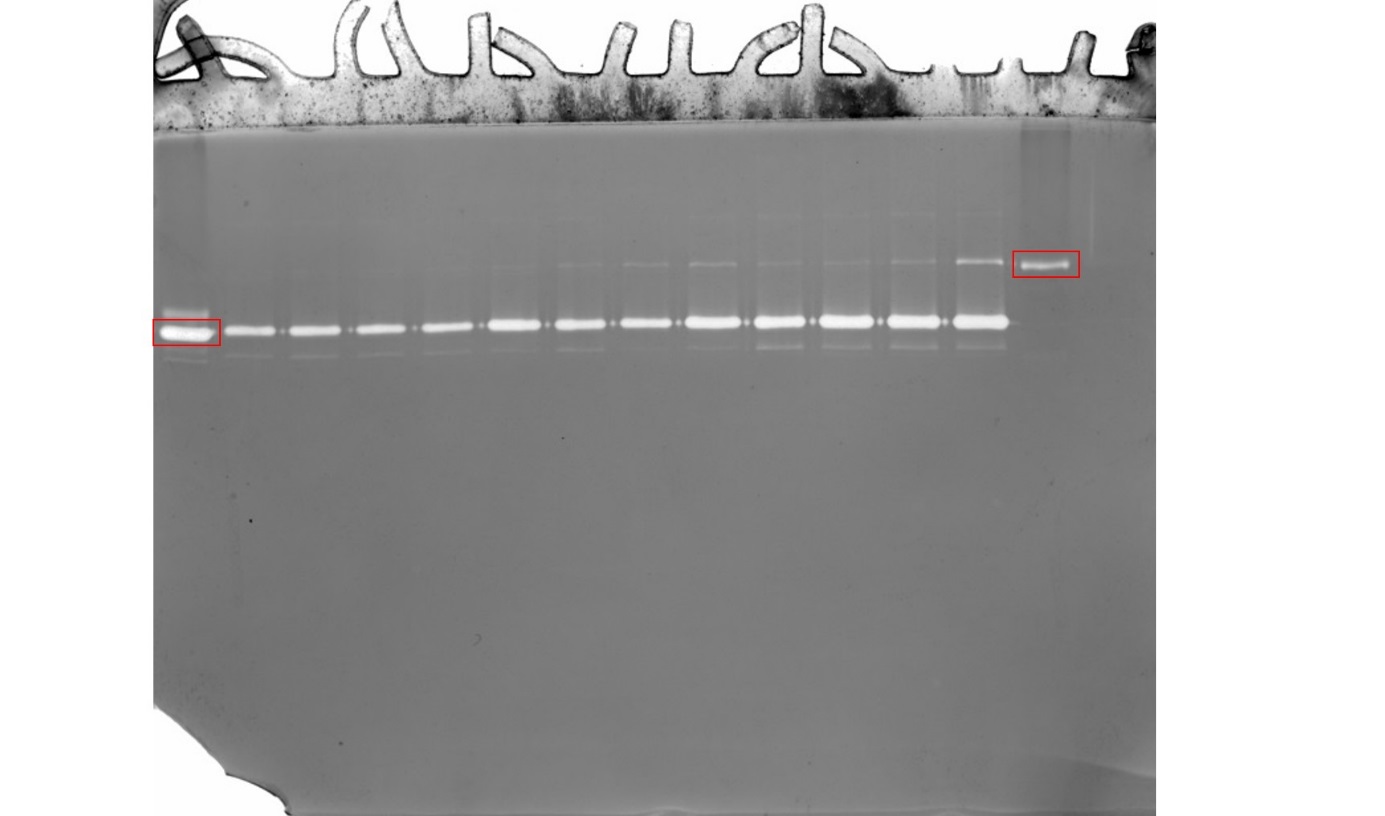
**

**Fig. S6.** Electrophoretograms for fibroblast cell culture no. 6 as above-mentioned scheme, band for markers for pro-MMP-2 and pro-MMP-9 are in red square, respectively. Loaded: 5 ug protein per well. Zymography was repeated because of low visibility of bands for pro-MMP-9. 25 ug protein per well was loaded. Results below.

**The scheme for electrophoretogram for fibroblast cell culture no. 4 (Fig. S7):**

- **Pro-MMP-2 marker**
- **Control 24h (well 2),**
- **TGF-β1 (1 ng/ml) 24 h (well 3),**
- **TGF-β1 (5 ng/ml) 24 h (well 4),**
- **TGF-β1 (10 ng/ml) 24 h (well 5),**
- **Control 48h (well 6),**
- **TGF-β1 (1 ng/ml) 48 h (well 7),**
- **TGF-β1 (5 ng/ml) 48 h (well 8),**
- **TGF-β1 (10 ng/ml) 48 h (well 9),**
- **Control 72h (well 1) (well 10),**
- **TGF-β1 (1 ng/ml) 72 h(well 11),**
- **TGF-β1 (5 ng/ml) 72 h(well 12),**
- **TGF-β1 (10 ng/ml) 72 h (well 13),**
- **Protein ladder,**

**
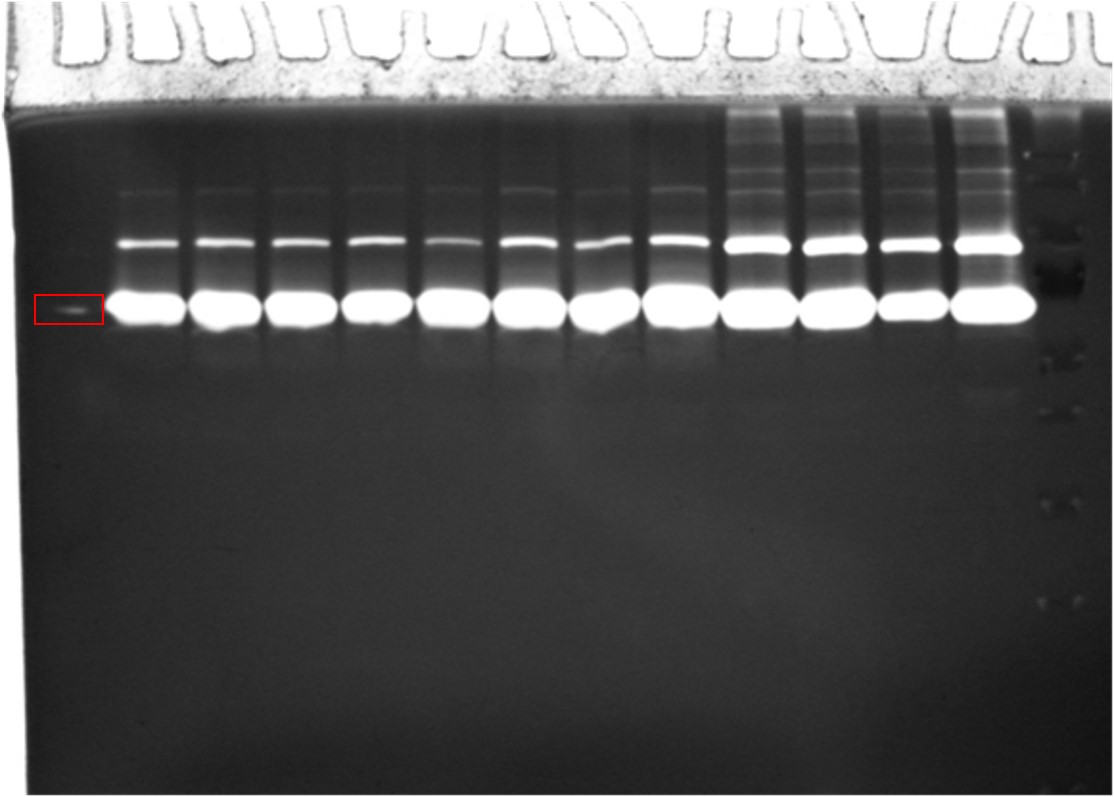
**

**Fig.S7.** Electrophoretograms for fibroblast cell culture 4 with treatment as above-mentioned scheme for pro-MMP-9. Band for markers for pro-MMP-2 is in red square Loaded: 25 ug protein per well.

**The scheme for electrophoretogram for fibroblast cell culture no. 6 (Fig. S8):**

- **Pro-MMP-9 marker**
- **Control 24h (well 2),**
- **TGF-β1 (1 ng/ml) 24 h (well 3),**
- **TGF-β1 (5 ng/ml) 24 h (well 4),**
- **TGF-β1 (10 ng/ml) 24 h (well 5),**
- **Control 48h (well 6),**
- **TGF-β1 (1 ng/ml) 48 h (well 7),**
- **TGF-β1 (5 ng/ml) 48 h (well 8),**
- **TGF-β1 (10 ng/ml) 48 h (well 9),**
- **Control 72h (well 1) (well 10),**
- **TGF-β1 (1 ng/ml) 72 h(well 11),**
- **TGF-β1 (5 ng/ml) 72 h(well 12),**
- **TGF-β1 (10 ng/ml) 72 h (well 13),**
- **Pro-MMP-2 marker**


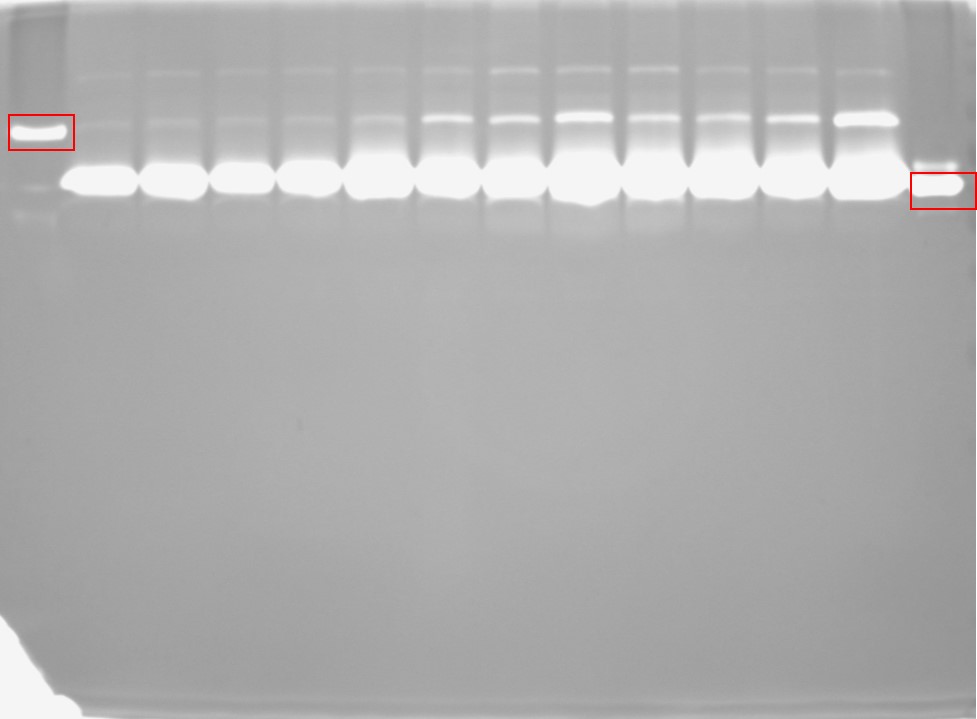


**Fig. S8.** Electrophoretograms for fibroblast cell culture no. 6 with treatment as above-mentioned scheme for pro-MMP-9 activity determination, band for markers for pro-MMP-9 and pro-MMP-2 are in red square, respectively. Loaded: 25 ug protein per well.
